# Case Series of Adenocarcinoma of the Prostate Associated with *Schistosoma haematobium* Infection in Tanzania

**DOI:** 10.4103/0974-777X.68540

**Published:** 2010

**Authors:** Humphrey D Mazigo, Maria Zinga, Jorg Heukelbach, Peter Rambau

**Affiliations:** *Departments of Medical Parasitology and Entomology, Weill-Bugando University College of Health Sciences, P.O. Box 1464, Mwanza, Tanzania*; 1 *Department of Community Health, School of Medicine, Federal University of Ceará, Fortaleza, Brazil*; 2 *Anton Breinl Centre for Tropical Medicine and Public Health; School of Public Health, Tropical Medicine and Rehabilitation Sciences, James Cook University, Townsville, Australia*; 3 *Department of Pathology, Faculty of Medicine, Weill-Bugando University College of Health Sciences, P.O. Box 1464, Mwanza, Tanzania*

**Keywords:** Adenocarcinoma, Prostate, *Schistosoma haematobium*, Schistosomiasis, Tanzania

## Abstract

In endemic areas, schistosomiasis has been associated with the pathogenesis of bladder, prostate, colorectal and renal carcinoma. However, the relationship between prostate cancer and schistosomiasis infection remains controversial. Here we present a series of three cases from Tanzania of prostatic adenocarcinoma associated with urinary schistosomiasis.

## BACKGROUND

Both urinary and intestinal schistosomiasis caused by *Schistosoma haematobium* and *Schistosoma mansoni* are endemic in many parts of Tanzania and pose serious public health problems.[1] According to the Tanzanian Ministry of Health, the prevalence of schistosomiasis in Tanzania varies from 13% to 88%, reaching 100% in some settings.

Studies have associated schistosomiasis with malignant neoplasia.[2] However, only few cases of co-occurrence of adenocarcinoma of the prostate and *S. haematobium* have been reported.[3,4] Here we document three cases from Tanzania of prostatic adenocarcinoma associated with *S. haematobium* infection.

## CASE REPORT

### Case 1

In 2009, a 50-year-old fisherman reported at Bugando Medical Centre (BMC), a referral hospital in the Lake Victoria Zone, with chief complaints of chronic hematuria and suprapubic pain of four months’ duration. The suprapubic pains were on and off radiating to the back. He reported dysuria but denied history of terminal hematuria, intermittent straining, constipation, fever, diarrhea or vomiting. He did not complain of any recent weight loss or chronic fatigue, and on physical examination there were no signs of emaciation. Clinical examination revealed suprapubic tenderness and a small suprapubic mass. Rectal examination revealed an enlarged, firm and slightly tender prostate.

In ultrasound examination, a mass was identified along the anterolateral walls of the urinary bladder, with irregular growth on the posterior wall of the bladder.

Laboratory investigations revealed a hemoglobin level of 8.3 g/dL; WBC count, 7800/mm^3^; and urine sediment >70 WBC and 5-10 RBC at high-power field; and a single egg of *S. haematobium*. A stool sample was negative for helminth infections.

Preoperative cystoscopy confirmed a growing mass at the urinary bladder neck in continuity with the prostate gland. A prostatectomy was then performed.

**Figure 1:**
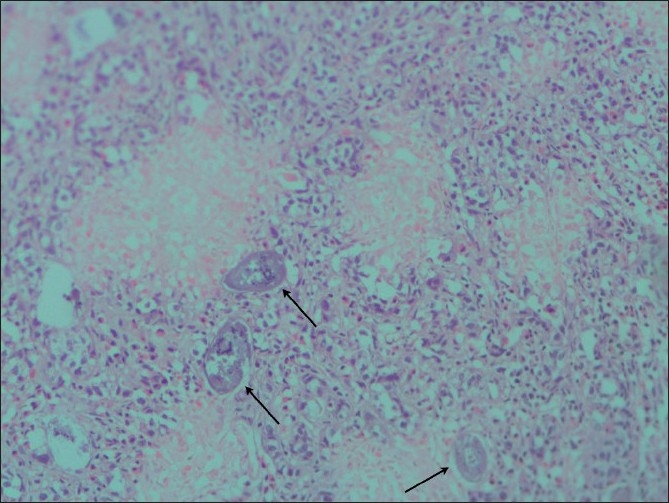
Histological section (hematoxylin and eosin stain) showing adenocarcinoma of the prostate. Arrows indicate eggs of *S. haematobium* (×100)

The surgical sample revealed a hard nodular prostate. Histopathological examination confirmed adenocarcinoma of the prostate [Figure 1] and a Gleason score of 8 (5+3). Scattered calcified *Schistosoma haematobium* eggs could be seen in the section. Acute and chronic inflammatory cells and fibrosis were present.

Schistosomiasis was treated with a standard single oral dose of praziquantel 40 mg/kg.

In the early postoperative period, the patient described no more suprapubic pain or dysuria, and urine was clean with no blood spots. He described only mild pain associated with the operation wound. The patient absconded from treatment after one week of admission and was then lost to further follow-up.

### Case 2

A 75-year-old man, a peasant, reported at the BMC in 2009 with complaint of frequent micturition more than ten times per day. Urine culture results showed a significant growth of coliform bacteria. There was no history of hematuria. Rectal examination revealed an enlarged and firm prostate gland. Ultrasound showed peri-calycal dilatation with thickened wall of the bladder, a dilated ureter and enlargement of the prostate gland. Cystoscopy revealed moderate bladder-wall irregularities. Urea and Hb levels were 36.19 mmol/L and 7.5 g/dL, respectively.

A trans-urethral resection of the prostate was performed, and grayish white prostatic chips were submitted to histopathology section for further analysis. Histopathological examinations confirmed adenocarcinoma of the prostate gland with a Gleason score of 7 (3+4). There were also fibrotic lesions and scattered *S. haematobium* eggs. The patient received a single dose of praziquantel (40 mg/kg) for treatment of schistosomiasis.

The patient was discharged after two weeks and was doing well on the first week of follow-up. The patient then did not return to avail of medical services and was lost to follow-up.

### Case 3

A 41-year-old man with a clinical history of frequent micturition reported a suprapubic mass present for one month. He denied any history of hematuria. Rectal examination revealed a huge mass in the anterior pelvic region. Ultrasound showed a diffuse hyperchromic mass in the distended urinary bladder, enlargement of the prostate gland with both kidneys showing a peri-calycal dilatation. Hb and urine creatinine levels were 5.3 g/dL and 55 μmol/L, respectively.

Prostatectomy and cystectomy were done. There was a bladder tumor involving the lateral wall, sparing only the posterior wall. Histopathological evaluation revealed adenocarcinoma of the prostate gland, infiltrations of inflammatory cells and scattered eggs of *S. haematobium*. The patient was referred to Ocean Road Cancer Institute, Dar es Salaam, Tanzania, for further treatment. There was no counter-referral from this medical center, and no further follow-up was possible.

## DISCUSSION

Genital involvement has been described to occur in infection with all species of *Schistosoma*, but it is particularly frequent in *S. haematobium* infection.[3] The adults of *S. haematobium* tend to deposit their eggs in the wall of the urinary bladder or, to a lesser extent, in the uterus, vaginal wall and prostate gland.[3] The immunological responses against miracidial soluble antigens lead to chronic cystitis associated with hyperplasia of the bladder mucosa and squamous metaplasia, and the resulting association of urinary schistosomiasis with bladder cancer is well established.[4]

On the other hand, the causal relationship between prostate cancer and schistosomiasis remains a matter of debate. It has been proposed that glandular atrophy associated with focal fibrosis of the prostate may lead to precancerous hyperplasia.[5,6] Other authors have suggested that the presence of nitrosamine carcinogens produced by nitrate-reducing bacteria and the enzyme beta-glucuronidase tend to act as cofactors for inducing neoplasia.[7]

The three cases presented in this case series are among the rare cases of coexistence of *S. haematobium* and adenocarcinoma of the prostate gland. Similar reports have been published from South Africa and Zambia.[8,9] Patients’ ages have been considered as an important factor for development of cancer associated with schistosomiasis. However, the association of *S. haematobium* eggs with adenocarcinoma of the prostate has been reported in young and older age groups.[8,9] Authors in these reports suggested that infections with large number of schistosome eggs at young ages could be one of the causal factors for development of prostate cancer.[8,9] The age at the time of diagnosis of prostate cancer in our patients is similar to that reported in these reports and another previous report.[8–10] In endemic areas, infections with *Schistosoma* species start at an early age, and severe manifestations of the disease are observed at adult ages.[1,8] It is possible that prostate tumors begin at early ages of the patients, at the time when schistosomiasis infections tend to occur in endemic areas.[8–10]

Unfortunately, all patients were lost to follow-up after a short period. The reasons for this are multifaceted and may include distance and access to the health services, as well as poverty. Many patients attending the referral hospitals come from resource-poor communities and are unable to pay for hospital bills. Thus they commonly ask for early discharge after surgery and do not return for follow-up examinations.

## CONCLUSION

We have reported three cases of adenocarcinoma of the prostate gland associated with schistosomiasis. The causal relationship between adenocarcinoma of the prostate gland and schistosomiasis remains unclear. In endemic areas, schistosomiasis should be considered as differential diagnosis for prostate gland enlargement. Further investigation is recommended on the possible causal relationship between the diseases in endemic areas.
